# Patterns of Dental Interventions Across Tooth Position, Age, and Sex: A Retrospective Real-World Clinical Study

**DOI:** 10.3390/healthcare14121637

**Published:** 2026-06-10

**Authors:** Bojana Milekić, Marko Štaka, Sara Koprivica, Đorđe Pržulj

**Affiliations:** 1 Faculty of Medicine, University of Novi Sad, 21137 Novi Sad, Serbia; bojana.milekic@mf.uns.ac.rs; 2 Faculty of Technical Sciences, University of Novi Sad, 21102 Novi Sad, Serbia; havzisara@uns.ac.rs (S.K.); przulj@uns.ac.rs (Đ.P.)

**Keywords:** dental symmetry, oral health epidemiology, FDI notation, retrospective study, tooth position, sex differences

## Abstract

**Highlights:**

**What are the main findings?**

- Dental interventions are overall bilaterally symmetric, with no significant left–right difference at the population level.
- Significant asymmetries occur in specific contexts, including the lower jaw, the 18–30 age group, and the first premolar position; however, these findings should be interpreted in the context of multiple comparisons.

**What are the implications of the main findings?**

- Findings support the use of symmetry as a baseline assumption in dental diagnostics, with attention to site- and age-specific deviations.
- Results can inform more targeted clinical protocols and population-level oral health strategies based on identified asymmetry patterns.

**Abstract:**

**Background/Objectives**: The lateral symmetry of dental disease and treatment patterns reflects underlying biological, behavioral, and systemic factors. Although clinical experience suggests asymmetric treatment frequencies, large-scale quantitative evidence remains limited. This study aimed to analyze the symmetry and asymmetry of dental interventions—encompassing restorations, extractions, endodontic treatments, and prosthetic procedures—in relation to tooth position, jaw, patient sex, and age group. **Methods**: A retrospective analysis was conducted on 30,063 dental intervention records from 2738 patients treated at a single private practice between 1999 and 2025. Tooth identification was based on FDI two-digit notation. Chi-square goodness-of-fit tests were applied to assess left–right and upper–lower symmetry, stratified by sex, age group, and tooth position. Bonferroni correction was applied to account for multiple comparisons (adjusted α = 0.0033). **Results**: Overall left–right symmetry was maintained across the full dataset (χ^2^ = 0.17, *p* = 0.682). After Bonferroni correction, only the right-side excess at the first premolar position remained statistically robust (*p* = 0.002), driven predominantly by the lower jaw (tooth 44 vs. 34; *p* = 0.011). The upper jaw received significantly more interventions than the lower jaw (56.3% vs. 43.7%, *p* < 0.001). Additional asymmetries observed at the uncorrected threshold—in the lower jaw (*p* = 0.026), in the 18–30 age group (*p* = 0.009), and a sex × jaw interaction (*p* = 0.047)—did not survive correction. **Conclusions**: Dental treatment patterns are predominantly symmetric at the population level. The most robust site-specific finding was a right-side excess of first premolar interventions, particularly in the lower jaw. These results may inform targeted preventive strategies and population-level oral health planning, with caution warranted in generalizing findings beyond the single-practice setting.

## 1. Introduction

Dental caries affects billions of people worldwide and remains one of the most prevalent chronic conditions across all age groups [1,2]. In 2021, approximately 2.37 billion cases of dental caries in permanent teeth were reported globally, with a higher burden observed in women than in men, according to the Global Burden of Disease (GBD) study [3]. Dental diseases, including caries, periodontal disease, and tooth loss, do not distribute randomly across the arch; they exhibit complex spatial distributions that have important clinical and epidemiological implications. Understanding whether these conditions and the interventions they necessitate distribute symmetrically or asymmetrically across the left and right sides of the dentition is of particular interest.

Caries prevalence is consistently higher in the maxillary jaw than in the mandibular jaw [2,4], with first and second molars carrying the greatest burden and mandibular central incisors the least affected. Premolars occupy a clinically important intermediate zone: the first premolar is a frequent target in orthodontic extraction planning, and its location at the boundary between the aesthetic and functional zones exposes it to differential occlusal loading and iatrogenic risk [2]. These positional patterns set the epidemiological context for the symmetry analysis conducted in this study.

Some prior studies have reported higher caries prevalence on the left side in right-handed individuals, hypothesizing that the dominant hand reaches the contralateral side more effectively during brushing, but these findings are inconsistent across the literature, and the effect, if it exists, is likely small [5,6]. Chewing-side preference may also contribute to asymmetric patterns of dental wear and pathology, as unilateral chewing has been shown to adversely affect hygiene status on the non-preferred side [7,8].

Age and sex both shape treatment patterns in clinically relevant ways. Younger patients present with orthodontic needs and developmental caries risks; older patients carry accumulated restorative histories that redirect clinical decisions independently of current disease [9]. Women show higher caries prevalence than men across diverse populations [10,11], while periodontitis is more severe in men [12]. Behavioral differences further compound these biological ones: men visit dentists less frequently and tend to present for acute problems rather than preventive care [13]. Socioeconomic factors also influence patterns of dental service utilization [14]. A 2025 systematic review and meta-analysis further confirmed that socioeconomic status is a significant predictor of dental service use among children and adolescents, with lower SES consistently associated with reduced utilization of both preventive and curative dental services [15]. Studies conducted in Serbia similarly indicate that individuals from socioeconomically disadvantaged backgrounds have greater oral health needs but paradoxically utilize dental services less frequently, thereby deepening oral health inequalities [16]. The age group of 18–30 years is of particular epidemiological interest because permanent dentition is complete, orthodontic extractions are at their peak frequency, and individuals are navigating oral health independently for the first time [9].

Tooth identification in this study follows the FDI two-digit notation system [17], in which the first digit identifies the quadrant and the second identifies the tooth position within the quadrant, numbered from the midline outward (Quadrant 1: upper right; Quadrant 2: upper left; Quadrant 3: lower left; Quadrant 4: lower right). This system is the internationally recommended standard for clinical and research documentation.

Data collected in a single dental practice over an extended period provide a methodologically specific perspective. Unlike population surveys, practice-based records reflect actual treatment decisions made under consistent clinical conditions over extended periods, yielding dense, internally coded datasets free from the inter-examiner variability that complicates multi-center studies. Their principal limitation—restriction to a specific patient population with particular socioeconomic and geographic characteristics—is a known and bounded source of bias.

The objective of this study was to analyze the symmetry and asymmetry of dental interventions—including restorations, extractions, endodontic treatments, and prosthetic procedures—recorded in a long-term single-practice database, examining the influence of tooth position, patient sex, and patient age group on bilateral treatment patterns. The study was guided by five hypotheses: (1) dental interventions are distributed bilaterally symmetrically at the population level; (2) the distribution of interventions differs between the upper and lower jaw; (3) age group modulates bilateral symmetry, particularly in younger patients; (4) the lateral distribution of interventions differs between male and female patients; and (5) specific tooth positions show position-specific deviations from bilateral symmetry.

## 2. Materials and Methods

### 2.1. Data Source and Study Design

This retrospective observational study used data extracted from the electronic patient management system of a single dental practice. The database encompassed records from 1999 to 2025 and included 2771 registered patients with 56,214 recorded intervention entries. The data were compiled and recorded primarily by a single practitioner throughout the observation period, which minimizes inter-clinician variability. No formal data quality audits were performed across the full 26-year period; this is acknowledged as a limitation of the study design.

The study was conducted in accordance with the principles of the Declaration of Helsinki and was approved by the Ethics Committee of the Dentistry Clinic of Vojvodina (approval number: 01-27/25-2026, date: 17 April 2026). Patient data were fully anonymized prior to analysis; no personal identifiers were retained in the analytical dataset.

### 2.2. Inclusion and Exclusion Criteria

Records were included if they contained a valid FDI tooth code corresponding to one of the 32 permanent teeth (positions 11–18, 21–28, 31–38, 41–48) [17]. Records with missing or invalid tooth codes, entries corresponding to primary dentition, and records with missing date of birth (preventing age calculation) were excluded. Interventions with dates outside the plausible range (before 1999 or after 2025) were also excluded.

After applying these criteria, 30,063 intervention records from 2738 patients were retained for analysis. The large discrepancy between the number of excluded patients and excluded intervention records reflects both the longitudinal nature of the dataset (some patients contribute a disproportionately large number of records over the 26-year observation period) and the fact that a majority of exclusions were applied at the record level (invalid FDI codes, primary dentition entries, or dates outside the plausible range) rather than at the patient level.

### 2.3. Variable Definitions

Dental interventions in this study encompass all recorded clinical procedures performed on permanent teeth, including restorations (fillings), tooth extractions, endodontic treatments (root canal therapy), and prosthetic-related procedures. Tooth position was defined according to the FDI two-digit notation. Side (left vs. right) and jaw (upper vs. lower) were derived from the quadrant designation.

Patient age at the time of intervention was calculated as the difference in days between the intervention date and the recorded date of birth, divided by 365.25. Age group was defined categorically: pediatric (<18 years), young adult (18–30 years), middle age (31–45 years), late middle age (46–60 years), and older adult (>60 years). These age boundaries were selected based on established clinical conventions: the threshold of 18 years corresponds to completion of permanent dentition and legal adulthood; 18–30 reflects the period of peak orthodontic activity and first-time independent oral health management [9]; 31–45 and 46–60 represent periods of progressive restorative accumulation; and >60 corresponds to demographic categories commonly used in geriatric oral health research [18].

Sex was recorded as Male (M) or Female (F) in the analytical dataset.

### 2.4. Statistical Analysis

Descriptive statistics were calculated for all variables. To assess bilateral symmetry, chi-square goodness-of-fit tests were used to compare the observed frequency of interventions on the right versus left side under the null hypothesis of equal (50%/50%) distribution. This was performed overall, and separately for each jaw, sex group, age group, and tooth position.

For contingency analyses (sex × jaw interaction, age group × side interaction), Pearson’s chi-square test of independence was applied. Statistical significance was set at α = 0.05. Given that more than fifteen chi-square tests were conducted across multiple strata, Bonferroni correction was applied to control for Type I error, yielding an adjusted significance threshold of α = 0.0033 (α = 0.05/15 tests). Both uncorrected and Bonferroni-corrected results are reported to allow direct comparison, and the interpretation of findings is based primarily on the corrected threshold. All analyses were performed using Python 3.12 with the SciPy library (version 1.11).

## 3. Results

### 3.1. Study Population

The final analytical sample comprised 30,063 dental intervention records from 2738 patients. The cohort was predominantly female (17,687 interventions, 58.8%) versus male (12,376 interventions, 41.2%). The mean patient age at the time of intervention was 44.5 years (SD = 16.9), with a median of 42.0 years and a range of 6 to 100 years. Demographic characteristics are summarized in Table 1.

### 3.2. Overall Lateral Symmetry

Across the entire dataset, 15,067 interventions were recorded on the right side and 14,996 on the left side (50.1% vs. 49.9%), demonstrating near-perfect bilateral symmetry (χ^2^ = 0.17, df = 1, *p* = 0.682). This result remained non-significant after Bonferroni correction and indicates no statistically significant overall preference for treating either side of the dental arch.

**Figure 1:**
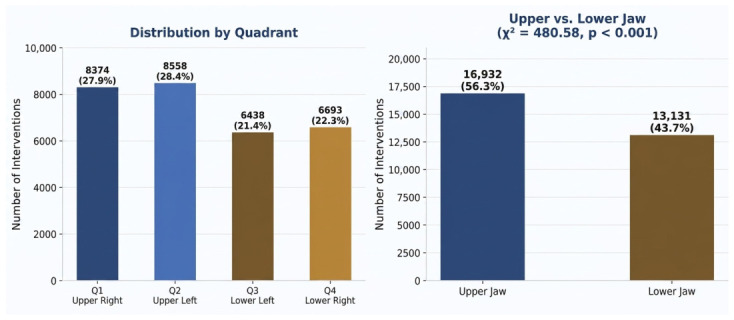
Distribution of dental interventions by jaw and quadrant.

Analysis by quadrant and jaw revealed that interventions are not distributed equally across the dental arches, as illustrated in Figure 1. Analysis by jaw revealed a significant asymmetry in the lower jaw at the uncorrected threshold: 6693 interventions were performed in the lower right quadrant (Q4) compared to 6438 in the lower left quadrant (Q3) (χ^2^ = 4.95, *p* = 0.026); however, this did not survive Bonferroni correction (α = 0.0033). The upper jaw showed no significant asymmetry (8374 right vs. 8558 left; χ^2^ = 2.00, *p* = 0.157).

A highly significant vertical asymmetry was observed: 56.3% of all interventions were performed in the upper jaw compared to 43.7% in the lower jaw (χ^2^ = 480.58, *p* < 0.001), a finding that remained significant after correction and is consistent with established epidemiological patterns. Results are presented in Table 2.

### 3.3. Lateral Symmetry by Sex

When stratified by sex, both male and female patients demonstrated symmetric treatment patterns. In male patients, 6148 interventions (49.7%) were recorded on the right side versus 6228 (50.3%) on the left (χ^2^ = 0.517, *p* = 0.472). In female patients, 8919 interventions (50.4%) were on the right versus 8768 (49.6%) on the left (χ^2^ = 1.289, *p* = 0.256). Neither result reached statistical significance.

A chi-square test of independence for the interaction between sex and jaw yielded a *p*-value of 0.047 (χ^2^ = 3.951), which is below the uncorrected α = 0.05 threshold but does not survive Bonferroni correction (α = 0.0033). This result should therefore be interpreted with caution as a preliminary finding requiring confirmation in independent, larger cohorts. Data are summarized in Table 3.

### 3.4. Lateral Symmetry by Age Group

**Figure 2:**
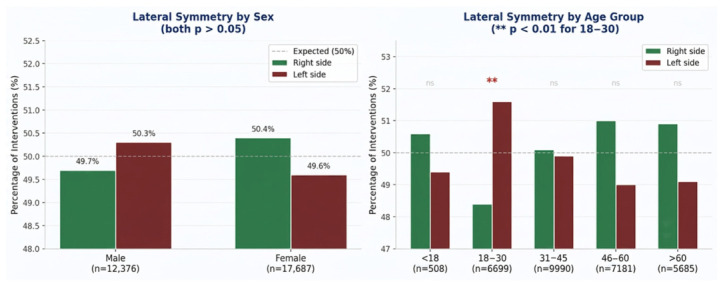
Lateral symmetry of interventions stratified by sex and age group. Dashed line = expected 50% distribution. ** *p* < 0.01, ns = not significant.

Analysis by age group revealed a statistically significant bilateral asymmetry in young adults aged 18–30 years at the uncorrected threshold: 3243 interventions (48.4%) were performed on the right side versus 3456 (51.6%) on the left (χ^2^ = 6.773, *p* = 0.009), indicating a left-side excess of 213 interventions (absolute difference: 3.2%). This result did not survive Bonferroni correction (α = 0.0033). All other age groups demonstrated symmetric distributions (all *p* > 0.05) (Figure 2).

A chi-square test of independence for the age group × side interaction was significant at the uncorrected threshold (χ^2^ = 11.764, df = 4, *p* = 0.019) but did not survive Bonferroni correction. It should be noted that the pediatric group (<18 years) comprised only 508 interventions (1.7% of the dataset), which represents limited statistical power and may explain the non-significant result (*p* = 0.790) for this group rather than confirming true symmetry. Full results are presented in Table 4.

### 3.5. Symmetry by Tooth Position

**Figure 3:**
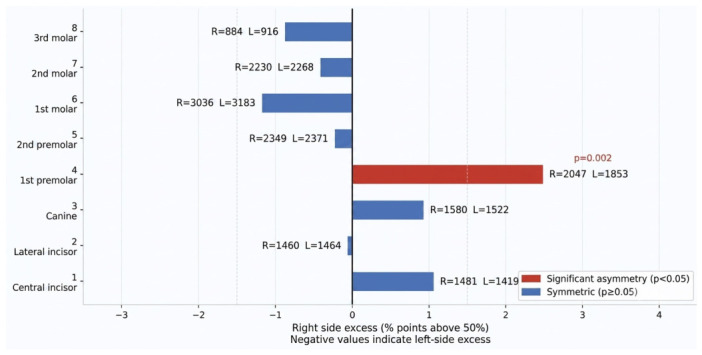
Deviation from bilateral symmetry by tooth position. Bars extending right indicate right-side excess; bars extending left indicate left-side excess. Red bar = statistically significant asymmetry (*p* < 0.05).

Analysis at the level of individual tooth positions revealed that seven of eight positions showed no statistically significant lateral asymmetry (all *p* > 0.05). The sole exception was position 4 (first premolar), which showed a significantly higher rate of right-side interventions (2047 right vs. 1853 left; R/L ratio = 1.105; χ^2^ = 9.650, *p* = 0.002). This result survived Bonferroni correction (α = 0.0033) and represents the most robust finding at the tooth-position level. The absolute difference was 194 interventions (4.98% of total first premolar interventions). The distribution of asymmetry across all tooth positions is illustrated in Figure 3.

**Figure 4:**
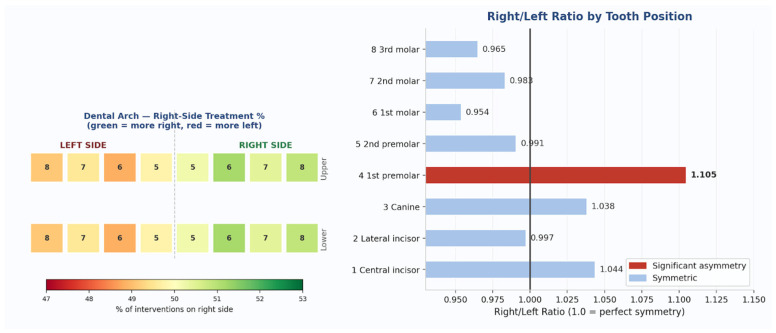
Visual overview of treatment asymmetry across the dental arch showing the dental arch heatmap (**left**) and R/L ratios (**right**).

Position 6 (first molar) showed a non-significant trend toward more left-side interventions (3183 left vs. 3036 right; χ^2^ = 3.475, *p* = 0.062); no inference of a directional trend is made from this result. The full spatial pattern of lateral asymmetry across the dental arch, including R/L ratios per position, is presented in Figure 4. Results are presented in Table 5.

To address the recommendation for jaw-stratified analysis, the first premolar was additionally analyzed separately for the upper and lower jaw. Results are presented in Table 6. The asymmetry was statistically significant in the lower jaw (teeth 44 vs. 34; χ^2^ = 6.406, *p* = 0.011) but did not reach significance in the upper jaw (teeth 14 vs. 24; χ^2^ = 3.441, *p* = 0.064), suggesting that the overall first premolar asymmetry is predominantly driven by the lower jaw, particularly tooth 44.

## 4. Discussion

This study provides a large-scale quantitative assessment of the lateral symmetry of dental interventions based on long-term clinical data. The main finding is that dental interventions are largely distributed symmetrically across the left and right sides of the dental arch at the population level, consistent with the known bilateral symmetry of dental anatomy. After applying Bonferroni correction for multiple comparisons, only the right-side excess at the first premolar (*p* = 0.002) survived as a statistically robust finding. The highly significant upper–lower jaw disparity (*p* < 0.001) also remained significant after correction and aligns with well-established epidemiological evidence.

The most robust position-level finding was the right-side excess of first premolar interventions (R/L ratio = 1.105, *p* = 0.002), which survived Bonferroni correction. Jaw-stratified analysis revealed that this asymmetry is predominantly driven by the lower jaw (tooth 44 vs. 34; *p* = 0.011), while the upper jaw showed only a non-significant trend (*p* = 0.064). The mechanism underlying this finding is not fully clarified by available data. One plausible explanation involves orthodontic treatment planning: the first premolar is the tooth most frequently selected for extraction in orthodontic cases requiring arch space [19]. However, standard orthodontic protocols most commonly involve bilateral (four-premolar) extractions [20,21], and asymmetric extraction protocols, while documented, are indicated only in specific malocclusion subtypes [22], which would not in itself produce a unilateral excess. The observed lower jaw right-side excess may instead reflect asymmetric occlusal loading patterns. Chewing-side preference, which affects a substantial proportion of the population, has been shown to influence the distribution of dental wear and potentially caries [7], and preferential right-side chewing in a predominantly right-handed sample could plausibly increase interventional demand on tooth 44. Iatrogenic factors—such as differential access and force application in the lower right quadrant—may also contribute but cannot be evaluated with available data. As the dataset does not include intervention type, these mechanistic explanations remain speculative, and future studies with procedure-stratified data are needed to resolve this question.

A left-side excess was observed in the 18–30 age group at the uncorrected threshold (*p* = 0.009, absolute difference = 213 interventions, 3.2%), but this did not survive Bonferroni correction. While statistically fragile, the directional pattern is noteworthy. Some prior studies have reported that right-handed individuals, who constitute approximately 88–90% of the population, tend to be less effective on the right side of the oral cavity during brushing due to the mechanical arc of hand movement [5,6], which could predispose the right side to greater caries accumulation and consequently more left-side interventions. However, the literature on this point is inconsistent [5], and the dataset contains no handedness data. An alternative interpretation involves the orthodontic extraction pattern described above: if asymmetric extractions are more prevalent in this age group and favor the left side in some cases, the left-side excess could partly reflect extraction asymmetry rather than differential disease susceptibility. Without procedure-type stratification, no causal interpretation can be confirmed.

The lower jaw lateral asymmetry (*p* = 0.026, absolute difference = 255 interventions, 1.9%) did not survive Bonferroni correction and therefore cannot be considered a robust finding of this study. As noted in the tooth-position analysis, the lower jaw asymmetry may be partly attributable to the first premolar finding (tooth 44), since the lower right quadrant contains this tooth. Demirci et al. (2010) similarly reported higher caries rates in specific quadrant–position combinations in a clinical database study [4]. This pattern is consistent with evidence from a recent systematic review and meta-analysis, which confirmed that caries distribution is generally symmetric between the left and right sides, while the maxillary teeth are more frequently affected than mandibular teeth overall, with specific positional variations across the dentition [23]. Future research could examine whether the lower jaw asymmetry in our dataset disappears after removal of first premolar records.

The highly significant upper–lower jaw disparity (56.3% upper vs. 43.7% lower, *p* < 0.001) is consistent with extensive prior epidemiological literature documenting higher caries prevalence and greater restorative need in the maxillary dentition [2,4]. Maxillary teeth, particularly molars and premolars, are known high-risk sites for interproximal and occlusal caries, and the maxillary anterior region frequently drives treatment-seeking for aesthetic reasons [24].

The sex × jaw interaction (*p* = 0.047) did not survive Bonferroni correction and should be treated as a preliminary, hypothesis-generating observation. With a sample of this size, even small deviations from zero can reach nominal statistical significance, and without an effect size measure this finding cannot be assessed for clinical relevance. If this interaction were to be confirmed in future multi-center studies, biologically plausible mechanisms would include hormonal influences on periodontal status—which disproportionately affects the lower jaw—or differential aesthetic motivation between sexes that might increase upper jaw treatment-seeking in female patients [12,13,25].

From a clinical significance perspective, it is important to note that even statistically significant asymmetries in this study involve modest absolute differences. The first premolar asymmetry represents 194 additional right-side interventions out of 3900 total (4.98%), and the 18–30 left-side excess is 213 interventions out of 6699 (3.2%). These magnitudes may not justify changes to individual clinical decision-making but could inform population-level resource allocation, preventive targeting, and the prioritization of screening for the lower right premolar region.

### Limitations

Several limitations must be acknowledged. First, data originate from a single private dental practice, limiting generalizability to broader or public-health populations. Second, and critically, dental interventions were not stratified by procedure type; restorations, extractions, endodontic treatments, and orthodontic procedures may show distinct symmetry profiles, and their pooling may obscure type-specific asymmetries. If stratification by intervention type were possible, it would likely substantially alter or clarify the observed patterns—this should be considered the most important limitation of the present analysis. Third, the dataset does not include information on handedness, dietary habits, or dental hygiene practices, all of which are relevant confounders. Fourth, more than fifteen chi-square tests were conducted; while Bonferroni correction was applied, this correction is conservative and may inflate the Type II error rate. Fifth, effect size measures (e.g., Cramér’s V or Cohen’s w) were not calculated for the statistically significant comparisons. Given the large sample size of this study, even small deviations from the null hypothesis can reach statistical significance; without effect size estimates, the practical clinical relevance of the identified asymmetries cannot be fully assessed. Sixth, the 26-year observation window introduces temporal confounding: secular trends in treatment philosophy, materials, and practice guidelines may have shifted the distribution of interventions over time. Seventh, although data were compiled primarily by a single practitioner, the long duration of the study and the possibility of inconsistencies in coding over time cannot be excluded. Eighth, the pediatric subgroup (<18 years) comprised only 508 interventions (1.7%), limiting statistical power for this group. Ninth, patients who attended the practice more frequently contributed disproportionately to intervention counts, meaning the unit of analysis is interventions rather than patients.

## 5. Conclusions

In this retrospective single-practice study of 30,063 dental interventions, lateral (left–right) symmetry was the dominant pattern at the population level. After Bonferroni correction for multiple comparisons, two findings survived as statistically robust: a right-side excess of first premolar interventions (*p* = 0.002), driven predominantly by the lower jaw (tooth 44), and a substantially higher intervention frequency in the upper jaw compared to the lower jaw (*p* < 0.001). Additional deviations observed at the uncorrected threshold—in the lower jaw, in the 18–30 age group, and as a sex × jaw interaction—should be regarded as preliminary observations requiring confirmation in independent, multi-center studies. The clinical magnitude of all identified asymmetries is modest. These findings may contribute to improved understanding of dental treatment distribution patterns and inform targeted preventive strategies at the population level, particularly with respect to the lower right first premolar region.

## Figures and Tables

**Table 1:** Demographic characteristics of the study sample.

Characteristic	Total	Male	Female	*p*-Value
	(n = 30,063)	(n = 12,376)	(n = 17,687)	
Sex distribution	100%	41.2%	58.8%	—
Mean age (years ± SD)	44.5 ± 16.9	44.5 ± 17.1	44.4 ± 16.7	0.714
Median age (years)	42.0	42.0	42.0	—
Age range (years)	6–100	6–100	6–100	—
Observation period	1999–2025	—	—	—

Note: *p*-value for age comparison between sexes derived from independent samples *t*-test.

**Table 2:** Distribution of interventions by jaw and quadrant.

Jaw	Q1 (Upper Right)	Q2 (Upper Left)	Q3 (Lower Left)	Q4 (Lower Right)	Total	χ^2^ (*p*-Value)
Upper jaw	8374 (27.8%)	8558 (28.5%)	—	—	16,932	2.00 (0.157)
Lower jaw	—	—	6438 (21.4%)	6693 (22.3%)	13,131	4.95 (0.026) *
Total (Right vs. Left)	15,067 (50.1%)	14,996 (49.9%)	—	—	30,063	0.17 (0.682)
Upper vs. Lower	16,932 (56.3%)		13,131 (43.7%)		30,063	480.58 (<0.001) ***

* *p* < 0.05; *** *p* < 0.001. Q1 = Upper Right, Q2 = Upper Left, Q3 = Lower Left, Q4 = Lower Right.

**Table 3:** Lateral symmetry of interventions stratified by sex.

Sex	Total (n)	Right Side	Left Side	R/L Ratio	χ^2^	*p*-Value
**Male**	12,376	6148 (49.7%)	6228 (50.3%)	0.987	0.517	0.472
**Female**	17,687	8919 (50.4%)	8768 (49.6%)	1.017	1.289	0.256
Sex × Jaw interaction	—	—	—	—	3.951	0.047 *

* *p* < 0.05.

**Table 4:** Lateral symmetry of interventions stratified by age group.

Age Group	n	Right (n/%)	Left (n/%)	R/L Ratio	χ^2^	*p*-Value	Sig.
<18 years	508	257 (50.6%)	251 (49.4%)	1.024	0.071	0.790	ns
18–30 years	6699	3243 (48.4%)	3456 (51.6%)	0.938	6.773	0.009	**
31–45 years	9990	5007 (50.1%)	4983 (49.9%)	1.005	0.058	0.810	ns
46–60 years	7181	3665 (51.0%)	3516 (49.0%)	1.042	3.092	0.079	ns
>60 years	5685	2895 (50.9%)	2790 (49.1%)	1.038	1.939	0.164	ns
Age group × Side	—	—	—	—	11.764	0.019	*

** *p* < 0.01; * *p* < 0.05; ns = not significant.

**Table 5:** Lateral symmetry of interventions by tooth position.

Position	Tooth Type	Right (n)	Left (n)	R/L Ratio	χ^2^	*p*-Value
1	Central incisor	1481	1419	1.044	1.326	0.250
2	Lateral incisor	1460	1464	0.997	0.005	0.941
3	Canine	1580	1522	1.038	1.084	0.298
4	1st premolar	2047	1853	1.105	9.650	0.002 ** †
5	2nd premolar	2349	2371	0.991	0.103	0.749
6	1st molar	3036	3183	0.954	3.475	0.062
7	2nd molar	2230	2268	0.983	0.321	0.571
8	3rd molar	884	916	0.965	0.569	0.451

** *p* < 0.01. † Only this result survived Bonferroni correction (α = 0.0033). FDI position: 1 = Central incisor, 2 = Lateral incisor, 3 = Canine, 4 = 1st premolar, 5 = 2nd premolar, 6 = 1st molar, 7 = 2nd molar, 8 = 3rd molar.

**Table 6:** Lateral symmetry of first premolar (position 4) stratified by jaw.

Jaw	Right (n)	Left (n)	R/L Ratio	χ^2^	*p*-Value
Upper jaw (14 vs. 24)	1023	941	1.087	3.441	0.064
Lower jaw (44 vs. 34)	1024	912	1.123	6.406	0.011 *
Combined (pooled)	2047	1853	1.105	9.650	0.002 **

* *p* < 0.05 (uncorrected). ** Neither result survived Bonferroni correction individually, but the combined result (*p* = 0.002) did.

## Data Availability

The data presented in this study are not publicly available due to privacy and ethical restrictions, as they originate from a private dental practice. Anonymized data may be made available from the corresponding author upon reasonable request.
